# Mitochondrial Phylogeography of the Proboscis Monkey (*Nasalis larvatus*) Reveals Riverine and Coastal Population Structure Across Eastern Borneo

**DOI:** 10.1002/ece3.74593

**Published:** 2026-10-30

**Authors:** Tri Atmoko, Widya Pintaka Bayu Putra, Maryatul Qiptiyah, Istiana Prihatini, Mukhlisi, Muhammad Azhari Akbar, Chika Yamada, Youdiil Ophinni

**Affiliations:** ^1^ Research Center for Biota Systems National Research and Innovation Agency Cibinong West Java Indonesia; ^2^ Research Center for Applied Zoology National Research and Innovation Agency Cibinong West Java Indonesia; ^3^ Technical Implementation Unit for Forest Tree Seed Region III Ministry of Forestry Yogyakarta Indonesia; ^4^ Research Center for Applied Botany National Research and Innovation Agency Cibinong West Java Indonesia; ^5^ Center for Southeast Asian Studies Kyoto University Kyoto Japan; ^6^ The Hakubi Center for Advanced Research Kyoto University Kyoto Japan

**Keywords:** colobine, conservation genetics, control region, genetic differentiation, Kalimantan, transboundary connectivity

## Abstract

The proboscis monkey ( *Nasalis larvatus* ) is an endangered colobine endemic to Borneo, largely confined to mangrove, peat‐swamp, and riverine forest and capable of swimming across the rivers and tidal channels that structure its habitat. How this riparian specialization influences the spatial distribution of genetic diversity across the species' range in Indonesia has not been characterized. We sequenced the mitochondrial control region from 32 individuals collected at 11 localities throughout North and East Kalimantan and combined them with published data from Sabah and Pulau Kaget. Twenty‐six haplotypes (Hd = 0.986; π = 0.029) are divided into three haplogroups, with their geographic distributions only partially separated. AMOVA revealed significant population differentiation (*Φ*_ST_ = 0.275, *p* = 0.018). Differentiation among localities was more accurately explained by least‐cost distance calculated over a combined riverine–coastal network than by straight‐line distance, supporting the idea that gene flow predominantly occurs along riparian and coastal corridors rather than crossing land. A haplotype found in Sembakung, Nunukan, and Sabah indicates mitochondrial continuity across the Indonesia–Malaysia border. The Pulau Kaget reserve population clusters with East Kalimantan rather than forming a separate southern lineage. Neutrality tests indicate a demographic expansion in the late Pleistocene within the most prevalent haplogroup. Calibrated *Simias*–*Nasalis* distances place the split between these sister genera in the Pleistocene (~0.74 Ma). These findings reveal regionally distinct and transboundary mitochondrial lineages, which should guide conservation efforts for this endangered primate.

## Graphical abstract

We sequenced the mitochondrial control region of 32 proboscis monkeys ( *Nasalis larvatus* ) at 11 localities across eastern Indonesian Borneo, the first phylogeographic survey of this endangered, river‐swimming colobine across eastern Indonesian Borneo. Genetic differentiation tracked least‐cost distance along rivers and the coast instead of straight‐line geography, and a haplotype shared across the Indonesia–Malaysia border showed that nationally separate populations belong to one genetic unit. We identify three clusters that should guide conservation management.

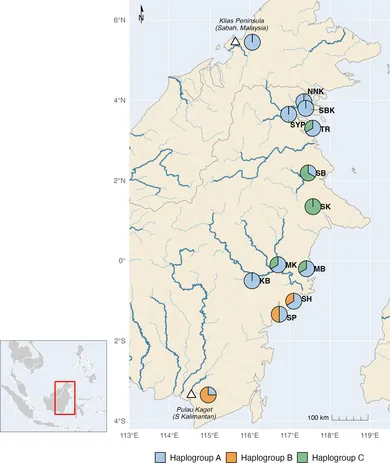

## 1 Introduction

In many vertebrates, including riparian and littoral mammals and freshwater and diadromous fishes, dispersal follows linear or branching landscape features (Dionne et al. 2008; Hughes et al. 2009; Mucci et al. 2010). Such features have two contrasting effects on differentiation among populations. Under a barrier model, the feature separates populations and differentiation tracks the strength of the barrier, as established for non‐swimming Amazonian primates (Ayres and Clutton‐Brock 1992; Helenbrook and Valdez 2025). In a corridor model, populations are confined to riparian or coastal habitats but can disperse freely along these areas. Differentiation then accumulates with distance along the network, rather than populations being separated at rivers. Rivers act as barriers for non‐swimming, terrestrial species but as corridors for strong swimmers that use the channel as habitat, such as the proboscis monkey ( *Nasalis larvatus* ).

The proboscis monkey (Figure 1) is endemic to Borneo and can be found in all four of its political regions: Indonesian provinces of Kalimantan, Malaysian states of Sabah and Sarawak, and Brunei. Its range is primarily in lowland habitats near water, including mangroves, nipa swamps, peat forests, and riverine forests, stretching from coastal areas to inland plains, usually following rivers and coastlines (Kawabe and Mano 1972; Bennett and Sebastian 1988; Meijaard and Nijman 2000; Phillipps and Phillipps 2016). As a foregut‐fermenting colobine, it mainly feeds on young leaves, unripe fruit, and seeds (Yeager 1989). It is the only primate in which regurgitation and remastication of stomach contents has been described—a behavior similar to rumination, linked to increased daily feeding time and more thorough mastication, enhancing food intake without compromising digestion (Matsuda et al. 2011). Groups are typically single‐male, multi‐female units that gather into bands at riverside sleeping sites, forming a multilevel social organization (Yeager 1990; Matsuda et al. 2024). Mating within groups is polygynous, with copulations usually single‐mount, mainly initiated by males but also by females (Murai 2006). Groups usually sleep in large emergent trees beside rivers, likely because these offer fewer mosquito bites and fewer access points for terrestrial predators (Feilen and Marshall 2014).

**FIGURE 1:**
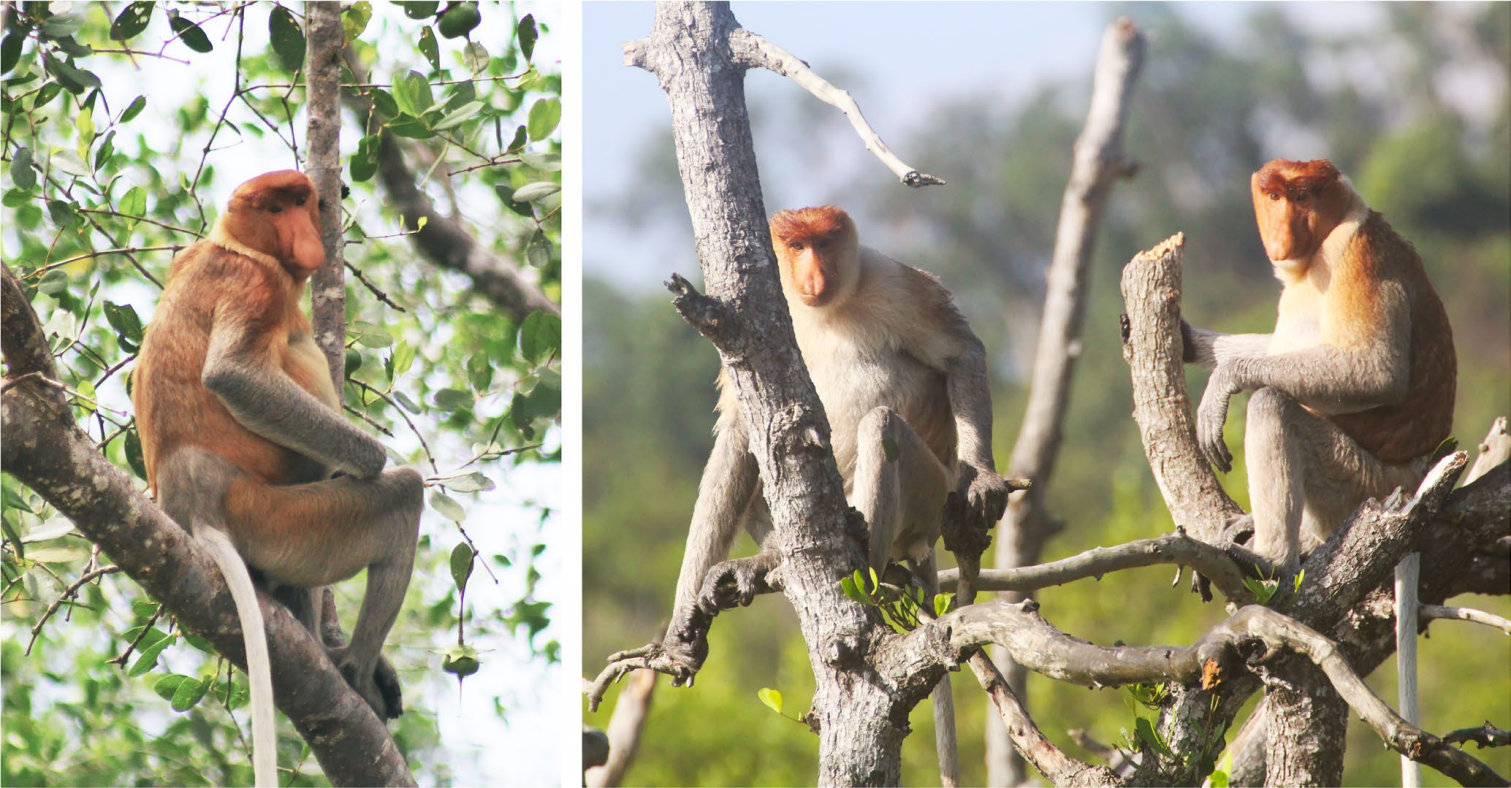
Adult *Nasalis larvatus* in East Kalimantan (photo by authors).

*Nasalis larvatus* is well‐adapted to crossing water, with partly webbed fingers and toes, and its home ranges often span both riverbanks, requiring swimming to access them (Yeager 1991). Such crossings are frequent, averaging 3.7 per group monthly at Tanjung Puting in Central Kalimantan (Yeager 1991), and almost daily across a tributary of the Kinabatangan River, about 25 m wide, with occasional crossings of the main river (Boonratana 2000). Crossings are not random; groups select narrower points (average width 13.5 m at crossing sites versus 17.5 m available), exhibit vigilance, coordinate crossings, and swim quickly, behaviors likely serving antipredator functions, especially considering crocodilians prey on monkeys at the water's edge (Galdikas 1985; Yeager 1991). The species also ranges close to water. Groups forage inland during the day but return to riverside trees to sleep, primarily using riverine forest and rarely entering inland dipterocarp forest (Galdikas 1985; Boonratana 2000). In one intensively studied group, ranges never exceeded 800 m from the riverbank (Matsuda et al. 2009). Both sexes disperse, females more widely than males, unlike in many cercopithecine primates (Matsuda et al. 2024). Consequently, an animal that crosses water easily but seldom moves far from it is expected to exchange genes primarily along the river and coastal network.

The species is also of conservation concern. It is classified as Endangered by the International Union for Conservation of Nature (IUCN; Boonratana et al. 2021), listed in CITES Appendix I (CITES 2021) and fully protected in Indonesia under Government Regulation No. 106/2018. Populations have declined over recent decades with habitat conversion across the range, driven by oil‐palm expansion, transmigration‐driven agriculture, infrastructure, and recurrent fires (Meijaard and Nijman 2000; Sha et al. 2008; Atmoko and Mukhlisi 2021; Toulec et al. 2022; Atmoko et al. 2024; Bernard, Abram, et al. 2025; Nijman and Shepherd 2025). Since 1973, Borneo has lost approximately 39% of its lowland forest and continues to experience significant annual deforestation (Gaveau et al. 2014), fragmenting these populations into more isolated demes (Atmoko et al. 2024). This fragmentation is likely to decrease gene flow, increase inbreeding within demes, and diminish the adaptive variation necessary to respond to environmental changes (Lino et al. 2019; Pinto et al. 2024). Whether this erosion is detectable in *N. larvatus* mtDNA, and at what scale, is unknown.

Mitochondrial DNA, and the rapidly evolving control region in particular (Pesole et al. 1999), has been a standard marker for genetic diversity and phylogeographic history across vertebrates, including primates (Avise 2000; Roos et al. 2014). In *N. larvatus* , prior sampling has been confined to Malaysian Borneo or to single reserve populations in southern Indonesia. Munshi‐South and Bernard (2011) characterized cytochrome *b* and control‐region variation in 70 individuals from Sabah and the Klias Peninsula; Ogata and Seino (2015) sequenced control‐region haplotypes from five captive Yokohama Zoo monkeys descended from Pulau Kaget founders translocated in 1997–1998 (Nijman 2016), and Mazlan et al. (2019) extended sampling in Malaysian Borneo using cytochrome oxidase I. The most comprehensive reanalysis drew mainly on samples from Sabah and South Kalimantan (Nijman 2016). Together, these studies indicate moderate to high diversity, haplotype sharing across Sabah subdivisions, and detectable substructure. Nevertheless, the published data come mostly from the Malaysian side and cannot determine whether genetic structure aligns with national boundaries. The wider distribution throughout eastern Indonesian Borneo, covering the Mahakam, Berau, Sembakung, Sesayap, and Kayan drainages and the connecting coastline, remains uncharacterized.

We sampled *N. larvatus* at 11 localities across North and East Kalimantan, Indonesia, including the major eastern Borneo drainages, and sequenced the mitochondrial control region from 32 individuals collected non‐invasively from feces. Our aim was, first, to characterize the spatial structure of mitochondrial diversity by examining whether differentiation among localities aligns with isolation by distance using Euclidean or network distance metrics. The second was to test whether Indonesian and Malaysian populations show genetic continuity across the border. The third was to examine the divergence between *N. larvatus* and its Mentawai sister taxon, *Simias concolor* , at an independent marker. Their relationship is taxonomically unsettled. Whittaker et al. (2006) found the cytochrome‐b divergence shallow enough to be compatible with placing *Simias* within *Nasalis*, in which case *Nasalis* would no longer be monotypic. Together, these provide the first phylogeographic characterization of *N. larvatus* across eastern Indonesian Borneo, clarify how Sundaland Pleistocene history and riparian habitat shape its diversity, and identify lineages relevant to regional conservation.

## 2 Materials and Methods

### 2.1 Sample Collection

Thirty‐two fecal samples, each from a different social group as judged in the field, were collected at 11 localities in eastern Borneo, Indonesia, including four locations in North Kalimantan Province and seven locations in East Kalimantan Province (Table 1). Fecal samples were collected from August to November 2015. Tarakan samples were collected within the *Kawasan Konservasi Mangrove dan Bekantan* (KKMB), a designated mangrove and proboscis monkey conservation area in Tarakan City.

**TABLE 1:** Sampling localities for *Nasalis larvatus* in eastern Borneo, Indonesia.

No.	Location	Province	Code	Coordinates	River system	*N*	*H*	Date of collection	GenBank accessions
1	Nunukan	North Kalimantan	NNK	3.96° N, 117.36° E	Sembakung‐Sebuku River system	3	2	20/11/2015	LC911870
2	Sembakung	North Kalimantan	SBK	3.80° N, 117.40° E	Sembakung River	3	3	22/11/2015	LC911858–LC911860
3	Sesayap	North Kalimantan	SYP	3.65° N, 116.98° E	Sesayap River	3	3	24/11/2015	LC911868–LC911869
4	Tarakan	North Kalimantan	TR	3.30° N, 117.58° E	Tarakan Island (coastal mangrove)	3	3	19/11/2015	LC911855–LC911857
5	Sungai Birang	East Kalimantan	SB	2.19° N, 117.46° E	Berau River	3	2	24/11/2015	LC911861–LC911862
6	Sungai Kelindakan	East Kalimantan	SK	1.35° N, 117.58° E	Kelindakan tributary, Berau system	3	2	20/11/2015	LC911874–LC911875
7	Muara Kaman	East Kalimantan	MK	0.10° S, 116.71° E	Mahakam River (lower)	3	3	17/08/2015	LC911863–LC911865
8	Muara Badak	East Kalimantan	MB	0.20° S, 117.42° E	Mahakam delta	3	3	20/11/2015	LC911871–LC911873
9	Kutai Barat	East Kalimantan	KB	0.50° S, 116.06° E	Mahakam River (upper)	3	2	12–15/08/2015	LC911876–LC911877
10	Sungai Hitam	East Kalimantan	SH	1.01° S, 117.10° E	Sungai Hitam (coastal tributary)	3	3	30/09/2015–02/10/2015	LC911878–LC911880
11	Sungai Parit	East Kalimantan	SP	1.34° S, 116.74° E	Sungai Parit (coastal tributary)	2	2	11–14/08/2015	LC911866–LC911867
					Total	32	26 unique*		

Abbreviations: *H*, number of unique mitochondrial control‐region haplotypes recovered; *N*, number of individuals sampled.

* Sum across localities (28) exceeds the global unique haplotype count (26) because two haplotypes recur at more than one locality: H18 (Table S1) is shared between Sembakung and Nunukan and is accessioned under SBK, and H23 is shared between Sungai Birang and Sesayap and is accessioned under SB.

Samples were preserved in RNAlater immediately after collection and stored frozen. Within each locality, samples were collected only from points more than 1 km apart and only where both deposits were fresh. Because a proboscis monkey group travels on average 910 m in a day (Boonratana 2000), two fresh deposits separated by more than this distance are unlikely to have been produced by the same group, and therefore by the same individual. Where field observation indicated that two deposits came from the same group, only one of them was collected.

### 2.2 DNA Extraction, PCR and Sequencing

The QIAamp DNA Stool Kit (Qiagen, Hilden, Germany) was used to isolate fecal DNA according to the manufacturer's protocol. Amplification of the mitochondrial control region was performed by PCR using primers NL2F (5′‐ACC CAA CAC CCA AAA TTG‐3′) and NL2R (5′‐TAA GAA CCA GAT GTC CGT‐3′) (Munshi‐South and Bernard 2011). DNA was extracted from 0.44–0.59 g of fecal material per sample. Yields ranged from 3.0 to 211.0 ng/μl and A260/A280 purity ratios from 1.28 to 1.91, values typical of non‐invasively collected fecal samples. Five extracts had purity ratios below 1.7, and three samples produced less than 10 ng/μl, but all 32 samples successfully amplified and generated clear chromatograms. Per‐sample data are detailed in Table S2. Negative controls for extraction and PCR were included in every batch and remained blank. Amplification and sequencing were not independently repeated.

Each reaction was carried out in a total volume of 50 μL containing 50 ng of DNA, 1× KAPA 2G Fast Ready Mix, 0.1 mg of BSA, 0.25 mM of each primer, and nuclease‐free water to volume. Amplification was run on a ProFlex PCR System. The PCR procedure consisted of denaturation at 94°C for 5 min, followed by 35 cycles of denaturation at 94°C for 40 s, annealing at 55°C for 30 s, primer extension at 72°C for 90 s, and a final extension at 72°C for 7 min. The PCR products were visualized using a 1.5% agarose gel run at 120 V for 60 min and then stained with ethidium bromide. The amplicons were partially sequenced using the BigDye Terminator v3.1 Cycle Sequencing Kit and the ABI PRISM 3730xl Genetic Analyzer (Applied Biosystems, MA, USA).

### 2.3 Sequence Processing and Alignment

Sequencing was conducted only in the forward direction due to cost considerations for a large number of low‐template fecal samples. Reads were processed in Geneious Prime v2024.0.7 and end‐trimmed using the Trim Ends tool with default settings (modified‐Mott algorithm, error probability limit 0.05, roughly a Phred quality score of 13). Any remaining ambiguous positions after trimming were clarified through visual inspection of the chromatogram, and singletons supported by a low‐quality peak were not accepted.

Analyses used two alignments. The 32 sequences generated here aligned to 465 bp, each contributing 464 bases, with two indel positions. Dataset 1 combined them with the published *N. larvatus* sequences long enough to span a common 430‐bp window, namely two of the 16 Sabah haplotypes (HQ260730 and HQ260736; Munshi‐South and Bernard 2011) and four sequences from captive‐born descendants of wild‐caught Pulau Kaget founders (AB896716–AB896719; Ogata and Seino 2015; provenance per Nijman 2016), together with the four colobine reference sequences described below. Dataset 2 was assembled to use the remaining Sabah sequences, which range from 317 to 414 bp. It combined the 32 study sequences, the four Pulau Kaget sequences, and 15 of the 16 Sabah haplotypes, trimmed to a 283‐bp window in which all 51 sequences have data. One Sabah haplotype (HQ260737) was excluded because its 19‐bp deletion would have required masking 20 of the 283 sites in every sequence. It falls within Haplogroup A, so its exclusion affects no conclusion. Dataset 2 was not used for phylogenetic or distance analyses, where missing terminal data would bias branch lengths and pairwise distances.

Four additional Asian colobine sequences served as ingroup comparators or outgroups: *Simias concolor* (JF293095), *Pygathrix cinerea* (JQ821841), *Rhinopithecus avunculus* (KX609232), and *Trachypithecus hatinhensis* (HQ149046). Segments of the control region homologous to the *N. larvatus* alignment were extracted from each reference record at these positions: *Simias concolor* JF293095, 15,494–15,917 (424 bp); *Pygathrix cinerea* JQ821841, 15,474–15,899 (426 bp); *Trachypithecus hatinhensis* HQ149046, 15,488–15,910 (423 bp); and *Rhinopithecus avunculus* KX609232, 89–515 (427 bp). The first three entries contain complete mitochondrial genomes, whereas the last one is a partial D‐loop sequence. Each segment is an exact match to its original record and was not further modified. The dataset was aligned with the L‐INS‐i algorithm in MAFFT v7.520 (Katoh and Standley 2013) and checked manually; uneven 3′ ends caused by length differences among reference sequences were trimmed. This produced Dataset 1, an alignment of 42 sequences and 430 bp in which every sequence spans the full window.

### 2.4 Phylogenetic Analysis

The nucleotide substitution model was chosen using ModelFinder (Kalyaanamoorthy et al. 2017) based on the Bayesian Information Criterion, as implemented in IQ‐TREE v3.1.1 (Minh et al. 2020); the preferred model was K3Pu + F + I + G4 (lnL = −1976.03). Phylogenetic analyses used Dataset 1, which includes the six published *N. larvatus* sequences and the four colobine reference sequences. Phylogenetic inference was performed with IQ‐TREE using 1000 ultrafast bootstrap (UFBoot) replicates (Hoang et al. 2018) and 1000 SH‐aLRT branch tests (Guindon et al. 2010). Clades were regarded as well supported if UFBoot values ≥ 95 and SH‐aLRT values ≥ 80.

Bayesian inference was performed using MrBayes v3.2.7 (Ronquist et al. 2012) with the GTR + I + G model, which includes the K3Pu + F + I + G4 model preferred by ModelFinder as a special case. We ran two independent analyses, each with four chains (one cold and three heated) for 10 million generations, sampling every 1000 generations and discarding the first 25% as burn‐in. Convergence was confirmed with an average split frequency standard deviation of 0.006 (below the required 0.01 threshold), all parameters having effective sample sizes of over 3500 (threshold 200) as checked with Tracer v1.7.2 (Rambaut et al. 2018), and the potential scale reduction factor (PSRF) values ranged from 1.000 to 1.001. *Trachypithecus hatinhensis* was designated as the outgroup, while *Pygathrix cinerea* , *Rhinopithecus avunculus* , and *Simias concolor* were included as additional ingroup colobines. The ML and Bayesian trees were compared, and posterior probabilities were mapped onto matching clades of the ML tree for visualization in R v4.4 using ggtree (Yu et al. 2017) and treeio (Wang et al. 2020).

### 2.5 Genetic Diversity and Population Differentiation

A median‐joining haplotype network (Bandelt et al. 1999) was constructed in PopART v1.7 (Leigh and Bryant 2015) from the 38 *N. larvatus* sequences of Dataset 1, with sampling locality assigned as the population trait. Default parameters (epsilon = 0) were retained. Haplotype diversity (Hd), nucleotide diversity (π), Tajima's *D* (Tajima 1989), and Fu's *F*_S_ (Fu 1997) were calculated using the pegas package in R (Paradis 2010), with Fu's *F*_S_ significance assessed by 10,000 coalescent simulations under the sampling formula (Ewens 1972). Pairwise *F*_ST_ values among the 11 sampled localities were computed using the estimator implemented in the hierfstat package (Weir and Cockerham 1984; Goudet 2005), treating each unique haplotype as an allele at a single locus. Analysis of molecular variance (AMOVA) (Excoffier et al. 1992) partitioning genetic variation between and within populations was performed in pegas using the number of pairwise nucleotide differences as the distance metric, with significance assessed by 10,000 permutations. Pairwise nucleotide differences were calculated using R with ape and pegas. The mismatch distribution was examined and summarized with Harpending's raggedness index; no demographic model was applied, and the inference of expansion is based on Fu's *F*_S_. Haplotype and nucleotide diversity and the numbers of segregating and parsimony‐informative sites were calculated for the 32 study sequences at their full length of 465 bp. Neutrality tests, the mismatch distribution, AMOVA, and pairwise *F*_ST_ used the same 32 sequences within the 430‐bp window of Dataset 1. Dataset 2 was used only for a complementary haplotype network and its diversity statistics.

### 2.6 Isolation by Distance

The hypothesis that genetic differentiation among populations increases with geographic distance (isolation by distance) was tested using Mantel correlation analysis (Wright 1943). Pairwise *F*_ST_ values were linearized as *F*_ST_/(1−*F*_ST_) (Rousset 1997), with negative *F*_ST_ values set to zero prior to linearization. Pairwise great‐circle (Haversine) geographic distances between localities were computed from GPS coordinates using the geosphere package in R (Hijmans 2024). Mantel tests with Spearman rank correlation were conducted using the vegan package with 9999 permutations (Oksanen et al. 2009) on both raw and log‐transformed geographic distances.

To determine if a network‐based geographic‐distance metric that reflects *N. larvatus* dispersal corridors better explains genetic differentiation than straight‐line geographic distance, we built a combined riverine and coastal network using HydroRIVERS v1.0 vector data (Lehner and Grill 2013) derived from the Australasia regional file, clipped to 108°–120° E and 5° S–8° N and supplemented with the Borneo coastline and major near‐shore island coastlines (> 50 km long) from Natural Earth (1:10 m administrative boundaries) via the rnaturalearth package (Massicotte and South 2023) in R. All geometries were projected to UTM Zone 50 N (EPSG:32650) in sf. Rivers and coastlines were snapped to a 100‐m grid to fill gaps from endpoints separated by less than that distance. River endpoints within 15 km of the coastline were then connected to the nearest coastline point. Because *N. larvatus* can disperse across coastal channels (Bennett and Sebastian 1988; Yeager 1990; Boonratana 1993, 1999), offshore island coastlines within 20 km of an adjacent landmass were connected through inter‐island swim edges. Two localities (Tarakan and Sungai Birang) were linked to the main network via manual bridge edges measuring 3.1 km and 1.5 km, respectively, with weights based on their true geodetic lengths (Figure S1). The resulting geometries were formed into a spatial network using the sfnetworks package (van der Meer et al. 2024). Pairwise least‐cost path distances among the 11 sampling localities were calculated as Dijkstra shortest paths through this network, using sfnetworks::st_network_cost(). Simple Mantel and partial Mantel tests with Spearman rank correlation comparing *F*_ST_ to riverine and Euclidean distance matrices were conducted using vegan::mantel() and vegan::mantel.partial() with 9999 permutations.

### 2.7 *Simias*–*Nasalis* Genetic Distance Comparison

To evaluate the phylogenetic affinity between *Simias concolor* and *N. larvatus* in the context of the sister‐taxon hypothesis (Whittaker et al. 2006), pairwise genetic distances among all 42 sequences of Dataset 1 were computed under three models—Tamura‐Nei (Tamura and Nei 1993), Kimura 2‐parameter (Kimura 1980), and uncorrected p‐distance—using the dist.dna function of the ape package with pairwise deletion of gaps (Paradis and Schliep 2019). Distances within and between *Nasalis* haplogroups were compared with *Simias*‐to‐*Nasalis* distances and with distances from *Nasalis* to the other odd‐nosed colobines (*Pygathrix* and *Rhinopithecus*) and the langur outgroup *Trachypithecus*. Approximate divergence times for the *Simias*–*Nasalis* split were estimated from mean Tamura‐Nei distances using a mitochondrial control‐region substitution rate of 9.883 × 10^−8^ substitutions/site/year, the value reported for the entire human control region (Soares et al. 2009) and applied here under the assumption of broadly conserved primate mitochondrial evolutionary rates.

## 3 Results

### 3.1 Sequence Variation and Haplotype Diversity

The 32 sequences resolved 26 haplotypes, a haplotype‐to‐individual ratio of 0.81 (Figure 2; Table 1). Haplotype diversity (Hd) was high at 0.986 ± 0.013 and nucleotide diversity (π) moderate at 0.029 ± 0.002, reflecting many haplotypes, most separated from their nearest neighbor by few substitutions. Of the 465 sites examined, 59 were segregating, and 43 were parsimony‐informative.

**FIGURE 2:**
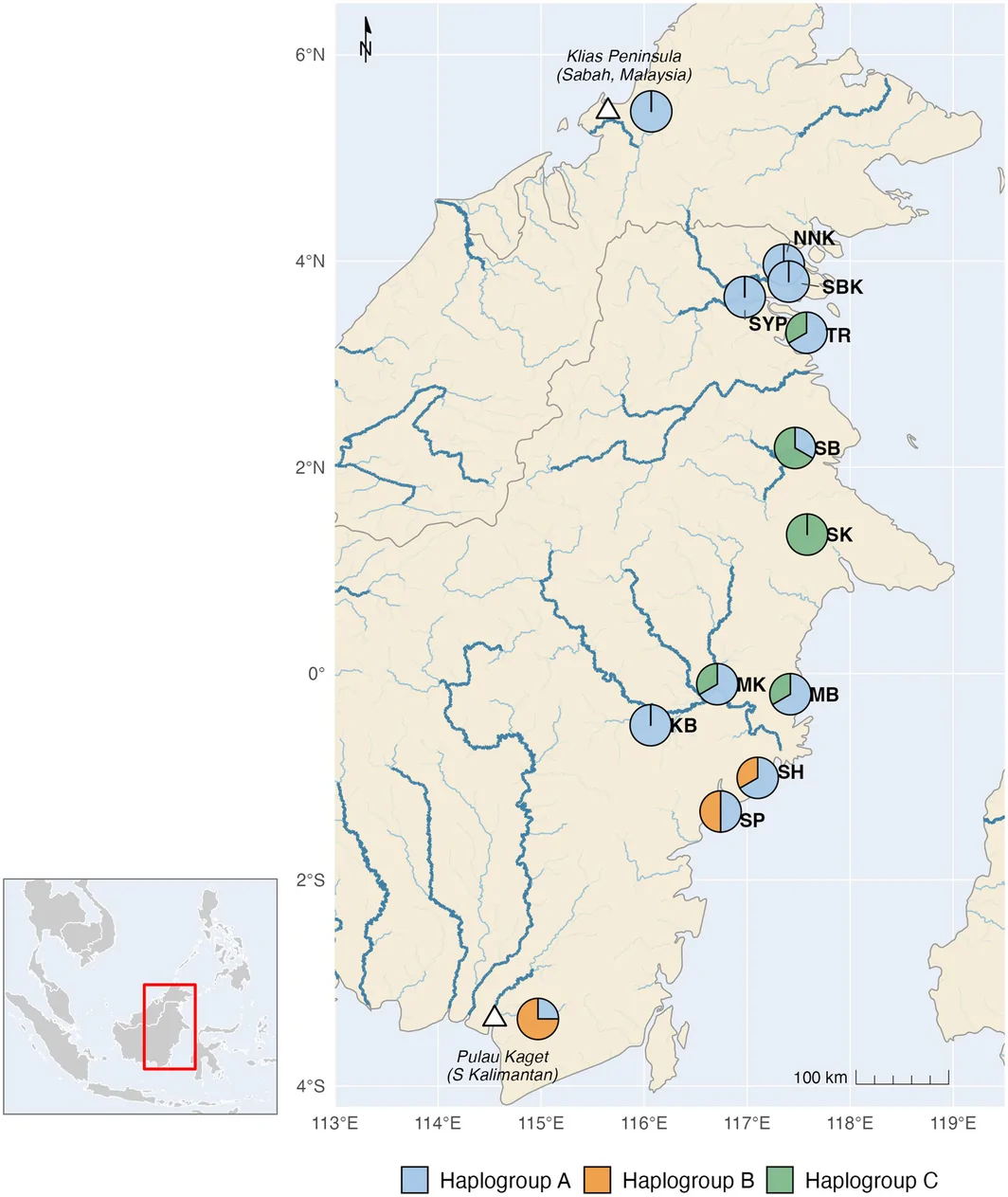
Sampling localities for *Nasalis larvatus* in eastern Borneo with haplogroup composition. Thirty‐two individuals were sampled at 11 localities across North and East Kalimantan, Indonesia (*n* = 2 at Sungai Parit, *n* = 3 elsewhere): NNK, Nunukan; SBK, Sembakung; SYP, Sesayap; TR, Tarakan; SB, Sungai Birang; SK, Sungai Kelindakan; MK, Muara Kaman; MB, Muara Badak; KB, Kutai Barat; SH, Sungai Hitam; SP, Sungai Parit. Pie charts show the proportions of the three control‐region haplogroups (A, B, C) at each study locality (Figure 4). Open triangles mark the two published reference localities, Klias Peninsula in Sabah (Munshi‐South and Bernard 2011) and Pulau Kaget in South Kalimantan (Ogata and Seino 2015), with adjacent pies showing the haplogroups of their published sequences (Sabah assignments from Dataset 2). Blue lines show Borneo's river network (HydroRIVERS v1.0; Lehner and Grill 2013), weighted by Strahler order: Trunk rivers (≥ 6) heavy, secondary (5) medium, tributaries (4) pale. Inset shows the panel location (red rectangle) within insular Southeast Asia.

### 3.2 Phylogenetic Relationships

Maximum‐likelihood inference in IQ‐TREE recovered the expected odd‐nosed colobine topology (with *Trachypithecus* as outgroup; *Pygathrix* sister to *Rhinopithecus*; that clade sister to the *Simias–Nasalis* clade; Figure 3). The Bayesian topology was congruent, with identical relationships among major lineages (Figure S2).

**FIGURE 3:**
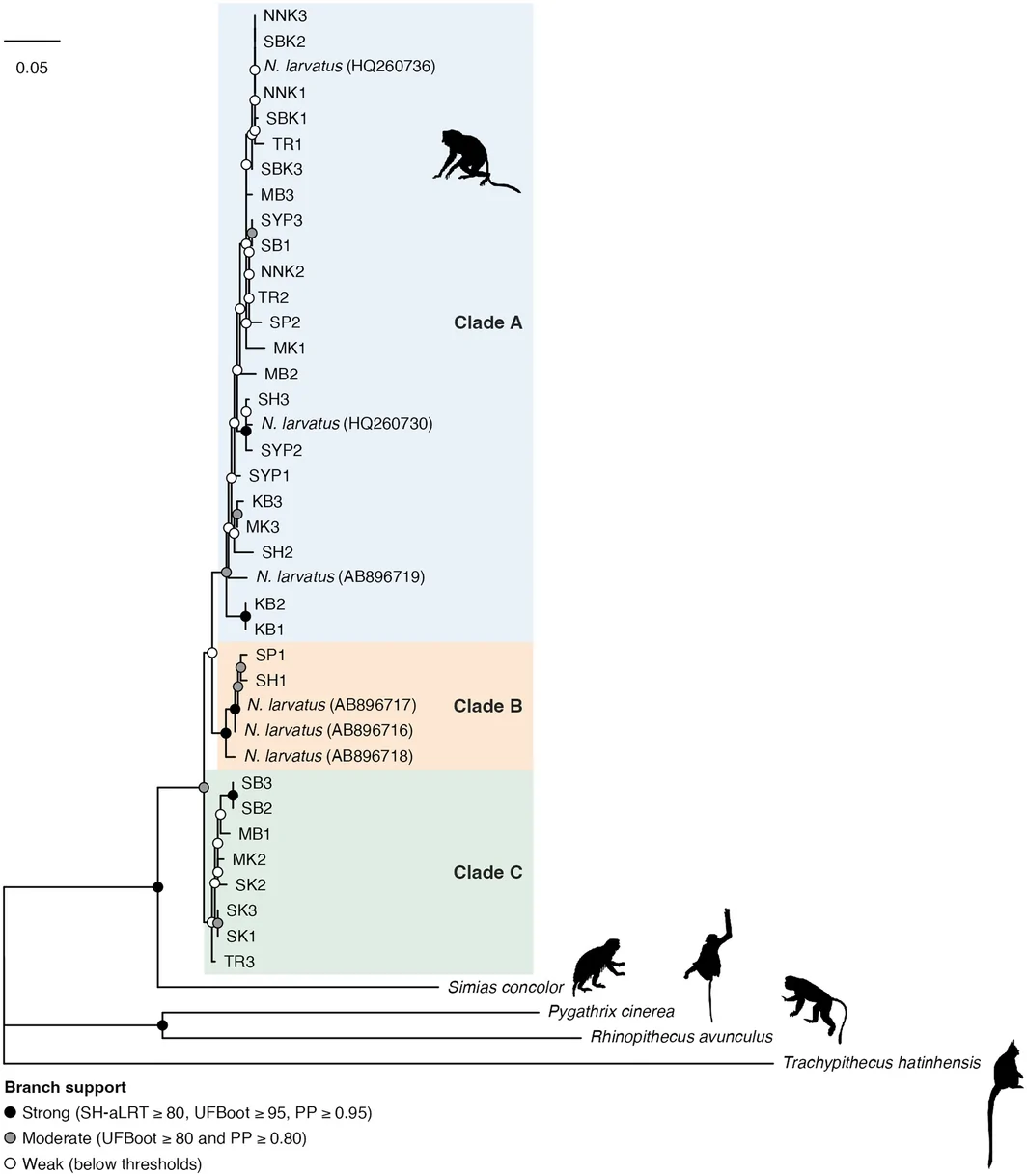
Maximum‐likelihood phylogeny of *Nasalis larvatus* control‐region haplotypes under the K3Pu + F + I + G4 model in IQ‐TREE v3.1.1 (lnL = −1976.03), rooted on *Trachypithecus hatinhensis* . Circles at internal nodes classify branch support from SH‐aLRT, UFBoot, and Bayesian posterior probability (PP), as defined in the key. Nodes absent from the Bayesian consensus are classified on UFBoot alone. Background bands mark the three *N. larvatus* clades (A, B, C). Reference sequences are *N. larvatus* from Sabah, Malaysia (HQ260730, HQ260736; Munshi‐South and Bernard 2011) and Pulau Kaget, South Kalimantan (AB896716–AB896719; Ogata and Seino 2015; provenance per Nijman 2016), with four other colobines: *Simias concolor* (JF293095), *Pygathrix cinerea* (JQ821841), *Rhinopithecus avunculus* (KX609232), and *T. hatinhensis* (HQ149046). Silhouettes (obtained from PhyloPic.org) are illustrative and show representative species within each genus instead of the reference taxa. The scale bar indicates substitutions per site.

The sister‐group relationship between *Simias* and *Nasalis* was strongly supported by both methods (SH‐aLRT = 82.3, UFBoot = 95, PP = 0.999). The branch separating the two genera was also shorter than the one separating *Pygathrix* from the *Simias*–*Nasalis* clade.

Three clades were identified within *N. larvatus* , with varying levels of support. Clade A is a large, diverse group including most haplotypes from East Kalimantan, North Kalimantan, and Sabah, together with one of the four Pulau Kaget sequences (AB896719); its internal nodes were mostly weakly supported. Clade B (PP = 0.99) is strongly supported and includes the other three Pulau Kaget sequences (AB896716–AB896718) and two haplotypes from East Kalimantan (SH1 from Sungai Hitam and SP1 from Sungai Parit), indicating unexpected genetic connectivity between South Borneo (Pulau Kaget) and the East Kalimantan coast. Clade C consists of six haplotypes (TR3, SK1, SK2, MK2, MB1, SB2) from eight individuals, five from the Berau drainage, two from the Mahakam (MK2 and MB1), and one from Tarakan (TR3). It has moderate ML support (UFBoot = 89) but weak Bayesian support (PP = 0.62), and diverges at the base of *N. larvatus* , sister to Clades A and B (PP = 0.81 for the node uniting all *N. larvatus* ). Relationships within Clade C are weakly supported, with most internal nodes below PP = 0.55. Mean p‐distance is 0.013 within Clade C and 0.041–0.047 between Clade C and the other clades (Table 4).

### 3.3 Haplotype Network and Geographic Patterns

The median‐joining haplotype network (Figure 4) recovers Clades A–C of the phylogenetic analyses as Haplogroups A–C. From north to south, the localities form a northern group in North Kalimantan (NNK, SBK, SYP, TR), a central group in the Berau drainage (SB, SK), and a southern group on the Mahakam River and the coast south of it (MK, MB, KB, SH, SP). Haplogroup A occurs in all three groups. Haplogroup C is concentrated in the Berau drainage, with single individuals at Tarakan, Muara Kaman, and Muara Badak, and Haplogroup B is confined to the two southernmost localities, Sungai Hitam and Sungai Parit. The three haplogroups form distinct clusters separated by 13–25 substitutions, consistent with TN93 distances of 0.043–0.050 between them.

**FIGURE 4:**
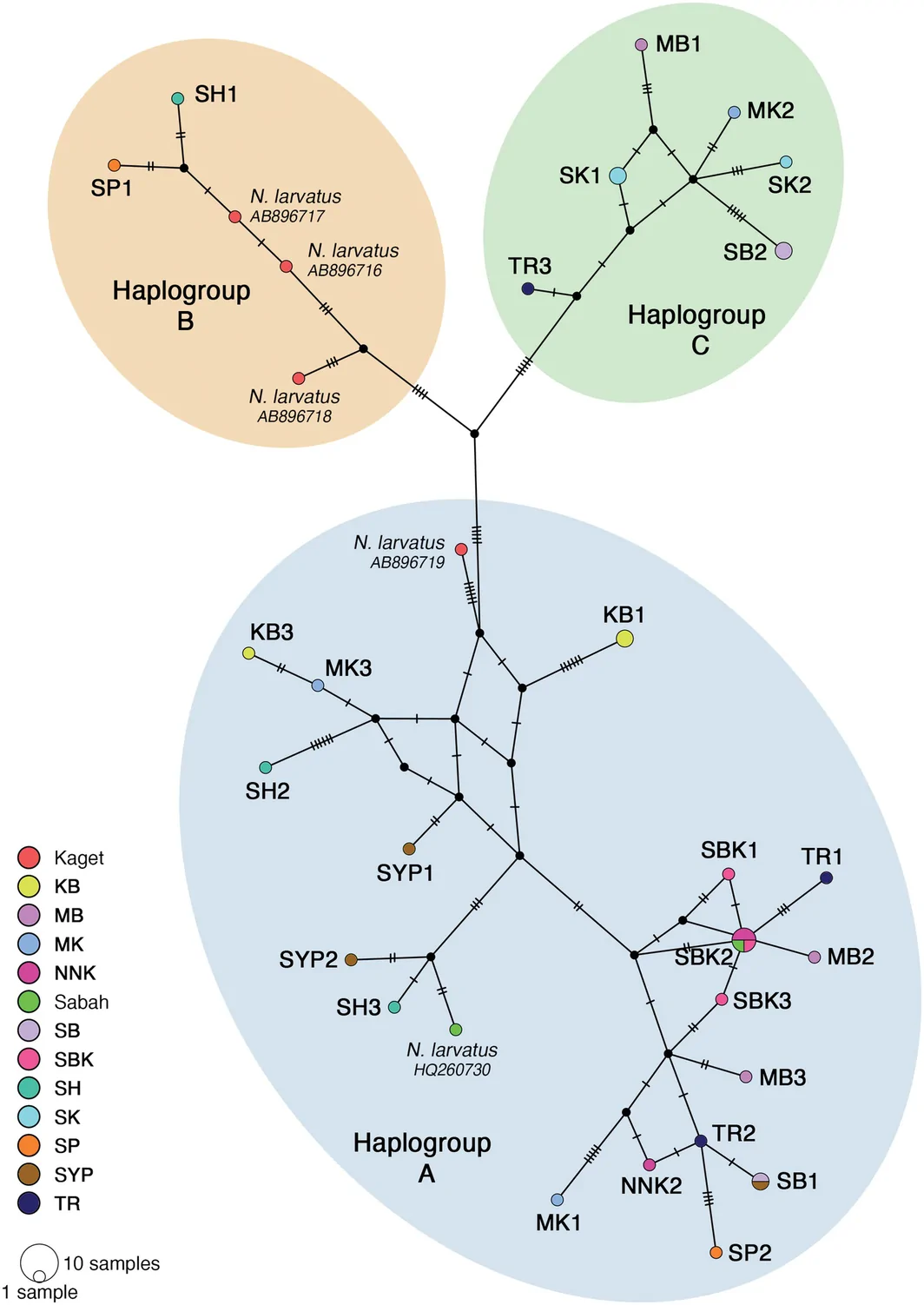
Median‐joining haplotype network for the *Nasalis larvatus* control region from the 38 sequences of Dataset 1, comprising the 32 study sequences and references from Sabah, Malaysia (HQ260730, HQ260736) and Pulau Kaget, South Kalimantan (AB896716–AB896719). Each circle represents a haplotype, with size corresponding to its frequency and color indicating its geographic origin. Ticks indicate nucleotide substitutions between neighboring haplotypes, while small black dots show inferred median vectors. The background ovals (pale blue, orange, green) illustrate the three haplogroups (A, B, C; Figure 3). The SBK2 haplotype, the most widespread in the network, is shared among Sembakung, Nunukan, and Sabah (HQ260736).

Five haplotypes were carried by more than one individual (Table S1), of which two recurred across more than one sampling locality. The most widespread haplotype, represented by sample SBK2, was shared among three localities—Sembakung (SBK), Nunukan (NNK), and Sabah (HQ260736; Munshi‐South and Bernard 2011)—spanning the Indonesia–Malaysia border. A second inter‐locality haplotype linked SB1 (Sungai Birang) and SYP3 (Sesayap). The remaining three recurrences were within‐locality replicates (SB2/SB3 at Sungai Birang, SK1/SK3 at Sungai Kelindakan, and KB1/KB2 at Kutai Barat).

Dataset 2, which includes 15 of the 16 published Sabah haplotypes, resolved 43 haplotypes among 51 sequences (Hd = 0.989, π = 0.040; Figure S3). All Sabah haplotypes fell within Haplogroup A. Haplogroups B and C were found only at Indonesian localities. The haplotype shared with Sabah (HQ260736) was found in four Indonesian individuals from Sembakung and Nunukan (SBK1, SBK2, NNK1, NNK3) at this shorter length, making it the most common haplotype in the network and the only one shared across countries. Restricted to the same 283‐bp window, the 32 study sequences give Hd = 0.980 and π = 0.042, close to the Dataset 2 values, so adding the published sequences does not alter the diversity estimates. Nucleotide diversity, although a per‐site measure, is higher in this window than across the full 465 bp (0.042 against 0.029) because the window spans a more variable part of the control region. Values from different windows are not interchangeable.

### 3.4 Population Differentiation and Geographic Isolation Over Distance

AMOVA placed 27.5% of the mitochondrial variation among the 11 sampled localities and 72.5% within them (*Φ*_ST_ = 0.275, *p* = 0.018; Table 2). Pairwise *F*_ST_ values (Weir and Cockerham 1984) ranged from −0.20 to 0.33 (Table 3). Negative values are sampling artifacts of the estimator and suggest effective panmixia. The greatest genetic differentiation was observed between Sungai Kelindakan (SK) and Kutai Barat (KB) with *F*_ST_ = 0.333, followed by Sungai Birang (SB) and Nunukan (NNK) with *F*_ST_ = 0.314. Conversely, some nearby northern localities such as Sembakung (SBK) and Nunukan (NNK) (*F*_ST_ = −0.020) showed signs of panmixia, indicating gene‐flow corridors that counter the broader regional structure.

**TABLE 2:** Analysis of molecular variance (AMOVA) on mitochondrial control‐region variation, showing distribution across 11 sampled localities of *Nasalis larvatus* in eastern Borneo.

Source of variation	df	Sum of squares	Variance components	% of variation	*Φ*‐statistic	*p* *
Among populations	10	1778.84	32.10	27.51	*Φ*_ST_ = 0.275	0.018
Within populations	21	1776.50	84.60	72.49		
Total	31	3555.34	116.69	100.00		

* Significance was assessed by 10,000 permutations.

**TABLE 3:** Pairwise *F*_ST_ values among the 11 sampled localities of *Nasalis larvatus* in eastern Borneo, with each unique haplotype treated as an allele at a single locus.

	NNK	SBK	SYP	TR	SB	SK	MK	MB	KB	SH
SBK	−0.020									
SYP	0.109	0.063								
TR	0.032	0.016	−0.078							
SB	***0.314***	***0.292***	−0.056	0.063						
SK	***0.250***	***0.250***	0.136	0.091	0.200					
MK	−0.034	−0.050	−0.078	−0.200	0.091	0.091				
MB	0.156	0.136	0.016	−0.065	0.129	0.100	−0.138			
KB	0.200	0.227	0.109	−0.111	***0.273***	***0.333***	−0.034	0.156		
SH	0.111	0.056	0.081	0.032	***0.250***	0.172	−0.071	0.069	***0.273***	
SP	0.239	***0.250***	0.026	−0.162	0.162	0.162	−0.053	0.039	0.068	0.202

*Note:* Localities are ordered from north to south along the eastern Borneo coast. Negative values reflect sampling noise. Bold‐italic cells indicate the highest pairwise *F*_ST_ values (≥ 0.25). Global structure was significant (AMOVA *Φ*_ST_ = 0.275, *p* = 0.018; see Table 2).

Linearized *F*_ST_ showed a positive but non‐significant correlation with Euclidean distance (Mantel *r* = 0.135, *p* = 0.110, 9999 permutations; Figure 5A). Substituting riverine + coastal‐network distance for Euclidean distance produced a stronger, near‐significant correlation (*r* = 0.190, *p* = 0.062; Figure 5B), and the regression slope was steeper. The two distance metrics showed a strong correlation (Mantel *r* = 0.827, *p* < 0.001), reflecting the meridional orientation of eastern Borneo's coastline. This collinearity limits how well partial tests can separate the contributions of the two metrics.

**FIGURE 5:**
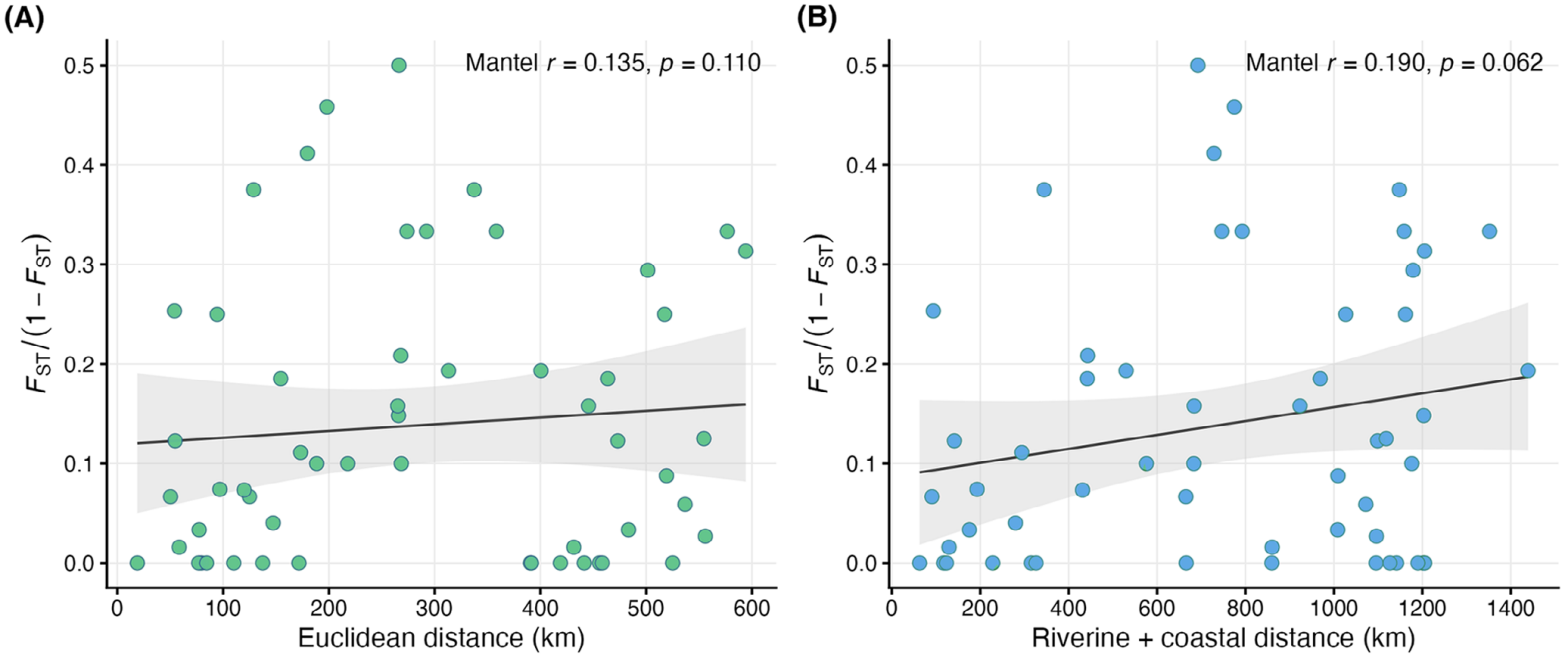
Isolation‐by‐distance plots of linearized *F*_ST_ [*F*_ST_/(1−*F*_ST_)] against (A) Euclidean great‐circle distance and (B) riverine + coastal‐network distance, for all 55 pairwise comparisons among the 11 *Nasalis larvatus* localities. Solid lines are linear regressions; shaded ribbons are 95% confidence intervals. Mantel correlations used Spearman rank correlation with 9999 permutations, treating each haplotype as an allele at one locus. Negative *F*_ST_ values (*n* = 14) were set to zero before linearization. The two distance metrics are strongly correlated (Mantel *r* = 0.827, *p* < 0.001), reflecting the meridional orientation of eastern Borneo's coastline.

Even so, after controlling for riverine distance, Euclidean distance retained no residual association with *F*_ST_ (partial Mantel *r* = −0.041, *p* = 0.538), whereas riverine distance retained a positive, though non‐significant, residual correlation when Euclidean distance was controlled (partial Mantel *r* = 0.141, *p* = 0.290).

### 3.5 Demographic History and *Simias*–*Nasalis* Divergence

Tajima's *D* for the eastern Borneo sample was −0.26 and not statistically distinguishable from zero (*p* = 0.79, normal approximation; *p* = 0.83, beta). Fu's *F*_S_, however, was −7.82 and highly significant (*p* = 0.0004 by 10,000 coalescent simulations), indicating an excess of haplotypes relative to the neutral expectation given the observed nucleotide diversity. The mismatch distribution was bimodal (Figure S4), with peaks near 8 and 20 differences. These correspond to comparisons within haplogroups (average of 8.08 differences, *n* = 260 pairs) and between haplogroups (average of 19.73 differences, *n* = 236), respectively. Consequently, the pooled distribution (Harpending's raggedness index = 0.0099) reflects the haplogroup structure described earlier, rather than the demographic history of any individual lineage, and we do not interpret it as evidence of expansion. In Haplogroup A, the most widespread and well‐sampled lineage, Fu's *F*_S_ was −6.38 (*p* = 0.0017). By calibrating the mean number of pairwise differences within Haplogroup A (8.40) using a mitochondrial control‐region substitution rate of 9.883 × 10^−8^ substitutions/site/year (Soares et al. 2009), we estimate the expansion occurred in the late Pleistocene, approximately 10^5^ years ago. We give only the order of magnitude because the estimate rests on a substitution rate estimated for humans and treats mean pairwise difference as a proxy for time since expansion.

Mean Tamura‐Nei distance between *S. concolor* and *N. larvatus* was 0.147 (range 0.131–0.163), approximately three‐fold greater than mean among‐haplogroup distances within *N. larvatus* (0.043–0.050). It was lower than distances separating *Nasalis* from other Asian colobines (mean 0.178 to *Pygathrix*; 0.206 to *Rhinopithecus*; 0.213 to *Trachypithecus*; Table 4). *Simias* and *Nasalis* were also closer to one another (0.147) than either was to *Pygathrix* (0.237 and 0.178, respectively). Calibrating the mean *Simias–Nasalis* distance against the Soares et al. (2009) substitution rate yields a divergence estimate of ~0.74 Ma (range 0.66–0.82 Ma across the observed pairwise Tamura‐Nei distances of 0.131–0.163).

**TABLE 4:** Mean pairwise genetic distances among *Nasalis larvatus* haplogroups, between *N. larvatus* and *Simias concolor* , and between *N. larvatus* and other Asian colobines.

Comparison	TN93	K2P	*p*‐distance
Within *Nasalis larvatus*			
Within Haplogroup A (*n* = 300 pairs)	0.021 (0.000–0.044)	0.021 (0.000–0.044)	0.020 (0.000–0.042)
Within Haplogroup B (*n* = 10 pairs)	0.012 (0.002–0.024)	0.012 (0.002–0.024)	0.012 (0.002–0.023)
Within Haplogroup C (*n* = 28 pairs)	0.013 (0.000–0.019)	0.013 (0.000–0.019)	0.013 (0.000–0.019)
All *N. larvatus* pairs (*n* = 703 pairs)*	0.034 (0.000–0.065)	0.034 (0.000–0.065)	0.033 (0.000–0.061)
Between haplogroups (within *Nasalis*)			
Haplogroup A versus B	0.045 (0.034–0.058)	0.044 (0.034–0.057)	0.042 (0.033–0.054)
Haplogroup A versus C	0.050 (0.034–0.065)	0.050 (0.034–0.065)	0.047 (0.033–0.061)
Haplogroup B versus C	0.043 (0.032–0.058)	0.043 (0.032–0.057)	0.041 (0.031–0.054)
Between *Simias* and *Nasalis*			
*Simias* versus Haplogroup A	0.147 (0.134–0.159)	0.145 (0.132–0.157)	0.128 (0.118–0.137)
*Simias* versus Haplogroup B	0.156 (0.153–0.163)	0.154 (0.151–0.161)	0.134 (0.132–0.140)
*Simias* versus Haplogroup C	0.143 (0.131–0.153)	0.141 (0.129–0.151)	0.125 (0.116–0.132)
*Simias* versus all *N. larvatus*	0.147 (0.131–0.163)	0.146 (0.129–0.161)	0.128 (0.116–0.140)
Between *Nasalis* and other colobines			
*Pygathrix* versus *N. larvatus*	0.178 (0.162–0.191)	0.176 (0.161–0.189)	0.155 (0.144–0.165)
*Rhinopithecus* versus *N. larvatus*	0.206 (0.184–0.222)	0.204 (0.183–0.219)	0.175 (0.160–0.186)
*Trachypithecus* versus *N. larvatus*	0.213 (0.193–0.227)	0.211 (0.192–0.225)	0.182 (0.168–0.192)
Pairwise reference distances			
*Pygathrix* versus *Simias*	0.237	0.234	0.197

* Pairwise distances were computed across all 38 *N. larvatus* sequences (32 study + 4 Pulau Kaget + 2 Sabah), yielding 703 pairs [C(38, 2)] for the “All *N. larvatus* pairs” row and the within‐haplogroup rows.

Using the same rate and expression for the average among‐haplogroup Tamura‐Nei distances, the divergence is about 0.23 Ma between Haplogroups A and B (range 0.17–0.29), 0.25 Ma between A and C (0.17–0.33), and 0.22 Ma between B and C (0.16–0.29). The mean coalescence within haplogroups is more recent: around 0.11 Ma for Haplogroup A, 0.06 Ma for B, and 0.07 Ma for C. All three haplogroups thus diverged during the middle to late Pleistocene, after the split from *Simias* and just before the expansion signal.

## 4 Discussion

We tested whether mitochondrial differentiation among proboscis monkey populations is better predicted by straight‐line distance or by least‐cost distance through a combined river–coastal network. Simple and partial Mantel tests both favored network over Euclidean distance, three haplogroups showed partially segregated distributions along the coast, and AMOVA recovered a significant *Φ*_ST_ of 0.275. These results favor a corridor over a barrier model of riverine and coastal gene flow.

### 4.1 Mitochondrial Diversity Comparison

Mitochondrial diversity across the 32 study sequences was high (Hd = 0.986). The haplotype‐to‐individual ratio of 0.81, against 0.23 in the larger Sabah sample, means that most animals we sampled carried a haplotype of their own, which reflects our broader geographic coverage. Our Hd also exceeds those of the two Asian colobines compared below, although that comparison rests on two species and a short fragment of the control region. In *Trachypithecus francoisi* , Liu et al. (2013) recovered a comparable Hd of 0.952 across 178 individuals from southern China and northern Vietnam, but found no signal of recent expansion (Tajima's D = 0.656; Fu's *F*_S_ = 2.872) and a Bayesian skyline indicating decline since ~50 ka. In the Yunnan snub‐nosed monkey *Rhinopithecus bieti* , Liu et al. (2007) resolved 30 haplotypes among 157 individuals with Hd of 0.944, and whole‐sample neutrality was non‐significant because of a deep mid‐Pleistocene split between clades. Only the most widespread clade in that study carried a significant Fu's *F*_S_ of −3.48, with an expansion dated to 0.16–0.05 Ma that is concordant with our timing. Our dataset has the same structure, and we likewise base the expansion inference on Haplogroup A alone (Fu's *F*_S_ = −6.38). Discrepancies between Tajima's D and Fu's *F*_S_ are common in mtDNA datasets of this size, often because *F*_S_ is more sensitive to recent expansions (Ramos‐Onsins and Rozas 2002).

### 4.2 Population Structure Across Eastern Borneo

Prior mitochondrial work on *N. larvatus* was confined to small areas or to other loci. Munshi‐South and Bernard (2011) sampled Sabah; Ogata and Seino (2015) sequenced captive Yokohama Zoo animals descended from Pulau Kaget founders translocated in 1997–1998 (Nijman 2016); and Mazlan et al. (2019) typed 50 individuals from Sarawak and Sabah at the cytochrome oxidase I (COI) locus. Reanalyzing the available sequences against GenBank references, Nijman (2016) recovered two mitochondrial clades, each containing both Indonesian and northern Bornean individuals. Our eastern Borneo sample extends this work. The SBK2 haplotype from Sembakung and Nunukan was identical to the Sabah sequence HQ260736, with a distribution spanning ~250 km and the international border. The Pulau Kaget population is less genetically isolated than its disjunct location suggests. Three of its four haplotypes are in Haplogroup B, matching East Kalimantan sequences from Sungai Hitam and Sungai Parit, while the remaining haplotype is in Haplogroup A with northern Bornean haplotypes, confirming Nijman's (2016) two‐clade pattern. Broader sampling also suggests that genetic structuring is influenced more by riverine and coastal connectivity than by straight‐line distances. Ogata and Seino (2015) suggested a north–south division between Indonesian and Malaysian populations based on four captive‐bred individuals from Pulau Kaget, when no wild Indonesian Kalimantan samples were available. Our data, which address this gap, do not support a straightforward partition based solely on national borders. They are also consistent with the shared Labuk Bay–Kalimantan COI haplotype observed by Mazlan et al. (2019).

The three haplogroups have overlapping geographic ranges. Haplogroup A is found throughout the sampled area, while Haplogroups B and C are mainly restricted to southern East Kalimantan and the Berau drainage, respectively. AMOVA and pairwise *F*_ST_ recapitulate this locality by locality; the Berau populations are differentiated from the northern and southern localities, proximate northern localities are effectively panmictic, and the Mahakam delta acts as a connectivity hub rather than a boundary. On a larger scale, Mazlan et al. (2019) found similar levels of COI subdivision (*Φ*_ST_ = 0.733, *Φ*_CT_ = 0.680) and identified the Lupar Line in southwestern Sarawak as a potential barrier. Although the two studies sample different parts of the island, both detect significant differentiation among localities with different markers. This structure coexists with a lineage, Haplogroup A, that spans the entire sampled range, so it reflects differences in haplogroup frequency among regions rather than fully separate populations. Of the two models outlined above, the corridor model is the more plausible for a fluent swimmer (Bennett and Sebastian 1988; Yeager 1990; Boonratana 1993, 1999), and matches Nijman's (2016) expectation that populations within a catchment are more closely related to one another than to adjacent catchments. The Haplogroup C haplotype at Tarakan, ~220 km from the Berau drainage, was unexpected under a strict catchment model. It may reflect long‐distance dispersal along the coastal mangrove corridor, retention of ancestral diversity at low frequency, or an undocumented introduction; larger Tarakan samples and nuclear markers would distinguish these.

### 4.3 Riverine and Coastal Connectivity as Gene Flow Pathways

Mantel tests support the corridor interpretation, which is expected from the species' ranging behavior. Groups return to riverside trees to sleep and cross rivers routinely at narrow points (Yeager 1991; Boonratana 2000; Matsuda et al. 2009). Females disperse more widely than males (Matsuda et al. 2024), so a maternally inherited marker tracks the more mobile sex, whose movement is channeled along the water rather than across the land between drainages. Network and Euclidean distances are highly correlated (*r* = 0.827), partly because of the meridional coastline, and this collinearity makes it impossible to formally reject the null hypothesis of the partial Mantel tests at α = 0.05. In Sabah, Matsuda et al. (2024) genotyped around 200 individuals at 23 microsatellite loci over a 4‐km section of the Menanggul River. They observed that pairwise differentiation among males increased with upstream–downstream distance. In their study and ours, genetic differentiation builds up with distance along the river, not across the land in between, at spatial scales two orders of magnitude apart and with different types of markers.

### 4.4 *Simias*–*Nasalis* Divergence

Our control‐region data support the cytochrome‐b findings of Whittaker et al. (2006) and the combined mitochondrial and nuclear analysis by Liedigk et al. (2012) that *Simias* and *Nasalis* are sister taxa, with the smallest divergence among the Asian colobine genera compared here. The distance between them is about three times the average among *N. larvatus* haplogroups. Calibrated with the control‐region rate of Soares et al. (2009), it corresponds to a split about 0.74 Ma, consistent with Pleistocene vicariance during the reorganization of Sundaland. Haplogroup A expanded much later, roughly 100,000 years ago. The split date aligns broadly with fossil‐calibrated *Nasalis*–*Simias* ages of 1.12 Ma from nuclear data and 1.92 Ma from mitochondrial data (Liedigk et al. 2012). The timing also fits the cyclical flooding and exposure of the Sunda Shelf, which historically connected and isolated Sumatra, Borneo, and the Mentawai Islands (Heaney 1991; Voris 2000). Liedigk et al. (2012) proposed that a now‐extinct Sumatran population of an ancestral lineage explains the Borneo–Mentawai distribution. Nuclear markers and fossil calibrations could refine these dates.

### 4.5 Conservation Implications

The high diversity we report reflects past demography and should not be read as a sign that populations are secure. Because differentiation accumulates along the river and coastal network, a population cut off from that network would not be easily replenished by dispersal across watersheds or coastlines. On the Klias Peninsula, *N. larvatus* populations declined by roughly 40% over 18 years while habitat cover changed by only 3.8%, which may be due to extinction debt from earlier habitat fragmentation (Bernard, Mohammad‐Shom, et al. 2025). Habitat suitability modeling points the same way as our genetic results. A maximum‐entropy analysis of the Berau Delta found riverine habitat to be the strongest predictor of suitability, contributing 60.9% to the model and confining suitable habitat to about 9% of the landscape (Atmoko et al. 2020). Habitat loss that breaks connectivity thus carries a high isolation cost. For example, an oil‐palm plantation across a riparian buffer or a port removing mangroves can isolate hundreds of kilometers of the network. A dryland conversion away from the network may also reduce habitat area, but it affects connectivity less. Continuous riparian forests along major waterways and extensive mangroves along coastlines therefore cannot be easily replaced by interior habitats.

The Berau populations, characterized by Haplogroup C at high frequency, contain a lineage not found elsewhere in our sample. The transboundary SBK2 haplotype calls for cooperation between Indonesian and Malaysian agencies, as separate national management divides a single population. Pulau Kaget, although geographically isolated, shares lineages with both the East Kalimantan coast (Haplogroup B) and northern Borneo (Nijman 2016), and may be better treated as a connected part of the population.

### 4.6 Limitations

Nuclear or genomic data would complement the mitochondrial signal by resolving the more restricted male‐mediated gene flow (Matsuda et al. 2024). Our collection criterion makes it unlikely that two samples came from the same animal, but individual identity was not confirmed with genotypic markers. Any duplication would matter little. It could only appear in the three within‐locality haplotype recurrences (SB2/SB3, SK1/SK3, KB1/KB2), and treating each as a single individual changes haplotype diversity from 0.986 to 0.990 and nucleotide diversity from 0.029 to 0.028, with 26 haplotypes remaining. Sequencing was forward‐only, which makes rare artifacts harder to tell from true variants than with bidirectional sequencing. Amplification and sequencing were not independently replicated, although negative controls remained blank, and key haplotypes are present in multiple individuals, not singletons. The Pulau Kaget origin of the four sequences from captive‐born animals relies on records of their founders; if those records are wrong, the affinity might reflect the true source population. With 11 localities and strongly collinear distance metrics (*r* = 0.827), our design resolves among‐region structure but cannot characterize fine‐scale isolation by distance within rivers or separate isolation by distance from isolation by resistance. Finally, a single, rapidly evolving non‐coding marker calibrated with a human‐based rate cannot establish a genus boundary, especially when compared to cytochrome b data, so our results neither confirm nor exclude the within‐genus classification suggested by Whittaker et al. (2006), and resolving this requires multilocus studies.

## 5 Conclusion

Our study identified riverine and coastal connectivity, rather than straight‐line distance, as the axis organizing mitochondrial diversity in *N. larvatus* . The Berau populations contain Haplogroup C at a high frequency, a lineage not found elsewhere in our sample and thus not replaceable from another group if lost. The northern populations share Haplogroup A with Sabah and live in habitat that crosses an international border, so they need management coordinated by both governments. Haplogroup B, present on the East Kalimantan coast and Pulau Kaget, is entirely within Indonesia, and its conservation depends on collaboration between provinces. All three are needed to preserve the species' mitochondrial diversity. Resolving this structure with nuclear markers and more extensive sampling is the priority for translating it into management.

## Author Contributions

**Tri Atmoko:** conceptualization (equal), funding acquisition (equal), investigation (equal), project administration (equal), resources (equal), supervision (equal), visualization (equal), writing – original draft (equal), writing – review and editing (equal). **Widya Pintaka Bayu Putra:** formal analysis (equal), methodology (equal), software (equal), validation (equal), writing – original draft (equal). **Maryatul Qiptiyah:** formal analysis (equal), investigation (equal), methodology (equal), writing – review and editing (equal). **Istiana Prihatini:** validation (equal), writing – review and editing (equal), investigation (equal), resources (equal), writing – review and editing (equal). **Muhammad Azhari Akbar:** writing – review and editing (equal). **Chika Yamada:** formal analysis (equal), methodology (equal), validation (equal), visualization (equal), writing – review and editing (equal). **Youdiil Ophinni:** formal analysis (equal), funding acquisition (equal), methodology (equal), project administration (equal), supervision (equal), validation (equal), visualization (equal), writing – original draft (equal), writing – review and editing (equal).

## Funding

This work was supported by the Ministry of Environment and Forestry of the Republic of Indonesia under DIPA Balitek KSDA 029‐07.2.649881/2015 to Tri Atmoko, and by the Hakubi Project of Kyoto University to Youdiil Ophinni. The funders had no role in study design, data collection and analysis, decision to publish, or preparation of the manuscript.

## Ethics Statement

Fecal sampling was approved by the Ministry of Environment and Forestry of the Republic of Indonesia (Permit No. 266/KSDAE‐SET/2015), upon recommendation from the Indonesian Institute of Sciences (LIPI) (No. 2124/IPH.1/KS.02.04/VIII/2015). All samples were collected non‐invasively from the ground, with no capture, handling, or disturbance of individual animals.

## Conflicts of Interest

The authors declare no conflicts of interest.

## Supporting information


**Figure S1:** River and coastal network among the 11 *Nasalis larvatus* sampling localities (red circles).
**Figure S2:** The consensus phylogeny of Nasalis larvatus mitochondrial control‐region haplotypes inferred.
**Figure S3:** Median‐joining network of the mitochondrial control region for 51 Nasalis larvatus sequences.
**Figure S4:** The distribution of pairwise nucleotide differences among 32 Nasalis larvatus control‐region.
**Table S1:** Distribution of mitochondrial control‐region haplotypes among the 11 sampled sites of *Nasalis larvatus* in eastern Borneo.
**Table S2:** Fecal sample mass, DNA yield, and purity for the 32 Nasalis larvatus samples from eastern.

## Data Availability

The 32 mitochondrial control‐region sequences from this study are accessible in GenBank (accession LC911855–LC911880). The sequence alignment, metadata, pairwise *F*_ST_ and geographic/network distance matrices, the haplotype‐by‐locality table, the constructed riverine–coastal network, and the R code for all analyses are stored in the Dryad Digital Repository (https://doi.org/10.5061/dryad.2jm63xt5n).
