# Supplementary material for: Mitochondrial Phylogeography of the Proboscis Monkey (Nasalis larvatus) Reveals Riverine and Coastal Population Structure Across Eastern Borneo
Source: Ecol Evol. 2026 Oct 30;16(11):e74593. doi: 10.1002/ece3.74593 (PMC13720759; doi:10.1002/ece3.74593)
Supplement: Supplementary file 1 [file ECE3-16-e74593-s001.pdf]

Supplementary files

Mitochondrial phylogeography of the proboscis monkey (*Nasalis larvatus*) reveals riverine and coastal population structure across eastern Borneo

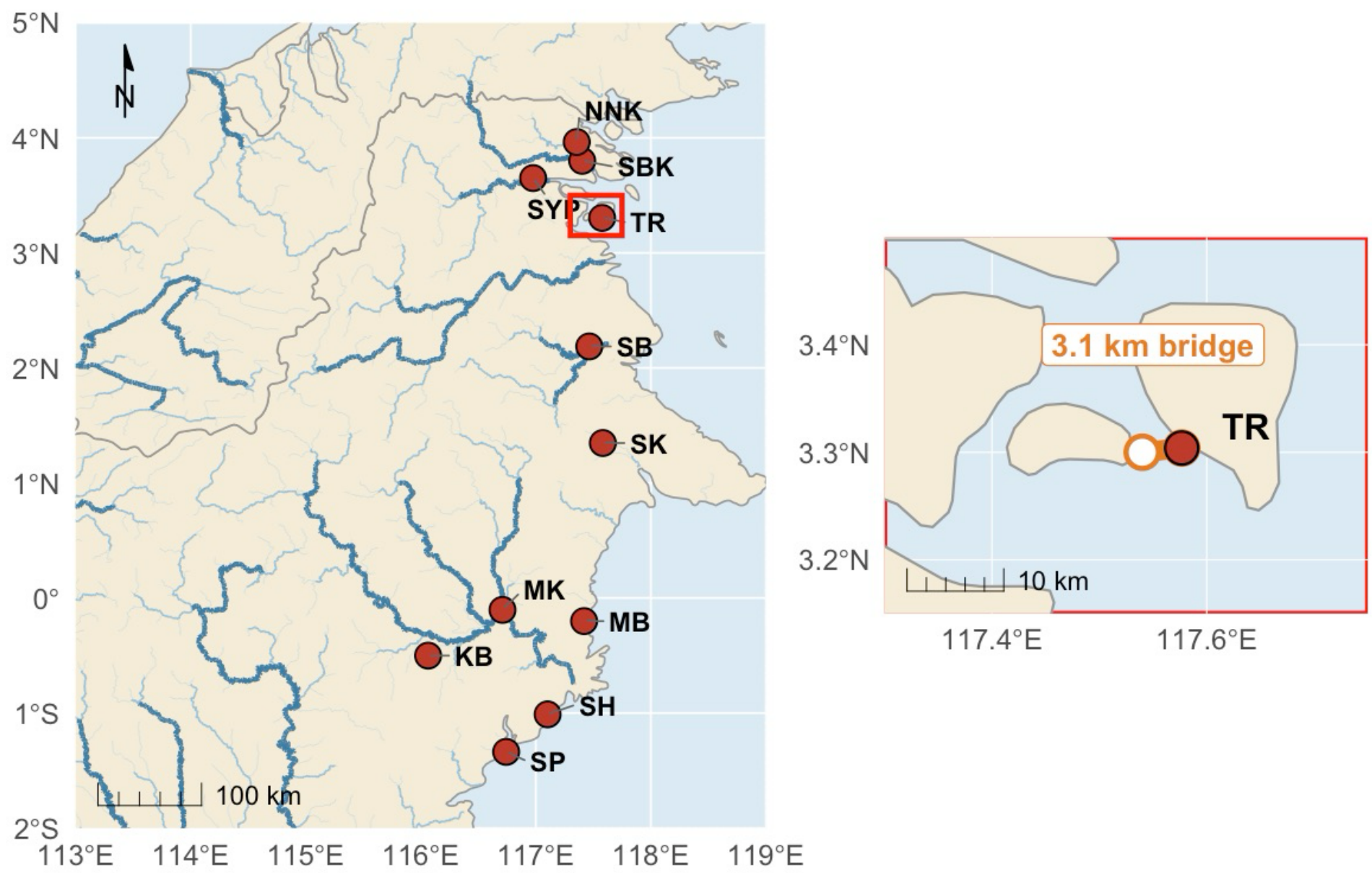

Fig. S1 River and coastal network among the 11 *Nasalis larvatus* sampling localities (red circles). **(Left)** Overview of eastern Borneo with its major river network (derived from HydroRIVERS) and coastline (from HydroSHEDS). Two bridges (orange lines with white endpoints) connect Tarakan Island (TR; 3.1 km bridge to the mainland) and Sungai Birang (SB; 1.5 km bridge to the nearest river segment). **(Right)** Inset close-up of Tarakan Island.

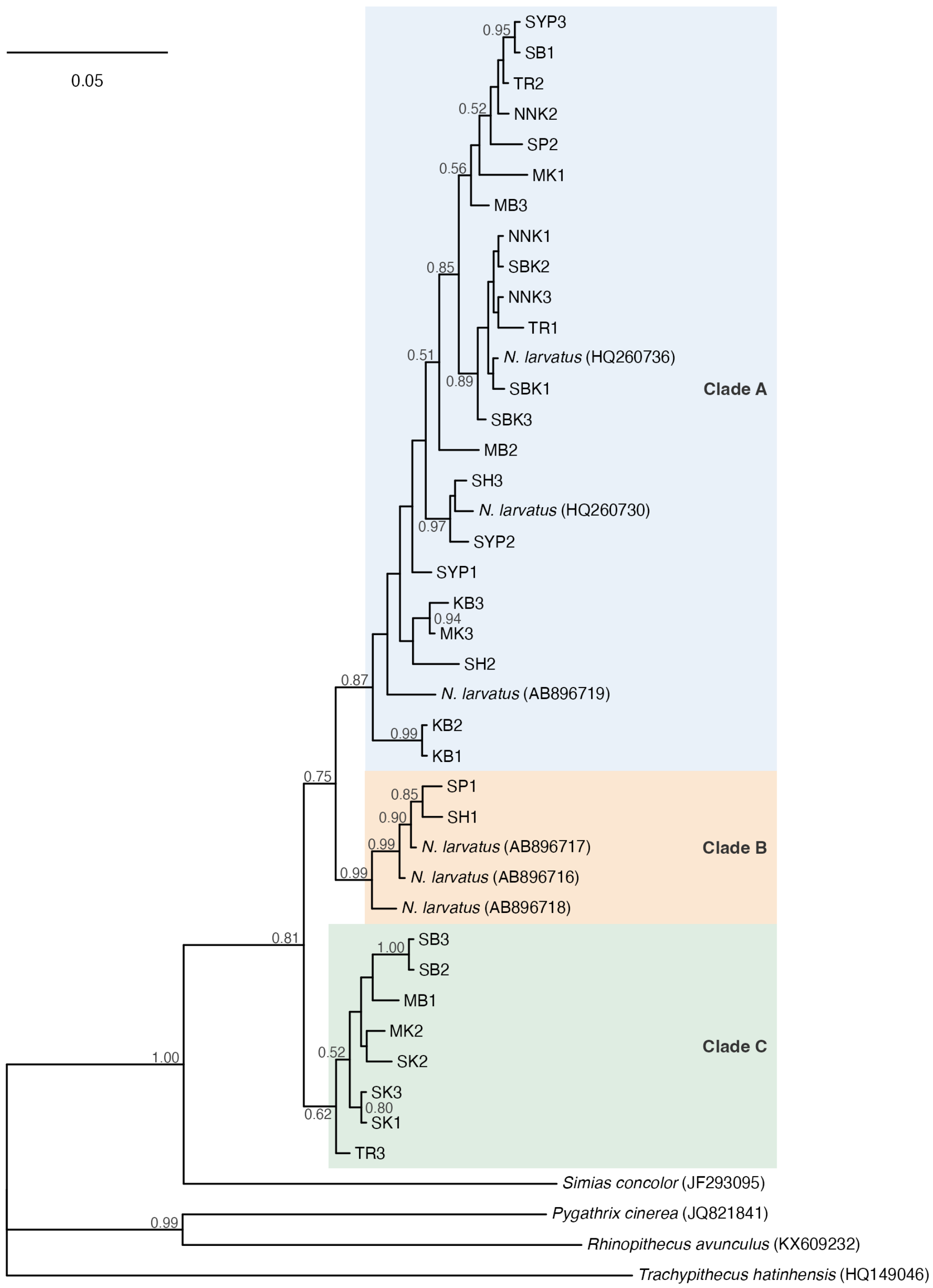

Fig. S2 The consensus phylogeny of *Nasalis larvatus* mitochondrial control-region haplotypes inferred using MrBayes v3.2.7 with the GTR+I+G substitution model. The analysis involved 10 million generations, with two independent runs and a 25% burn-in. Bayesian posterior probabilities are shown at the internal nodes, with only values ≥ 0.50 displayed. The tree is rooted on *Trachypithecus hatinhensis* (HQ149046). Three clades within *N. larvatus* (A, B, C) are distinguished by background color. The scale bar indicates substitutions per site. The average standard deviation of split frequencies is 0.006; all parameters' ESS exceeds 3,500; and the PSRF ranges from 1.000 to 1.001.

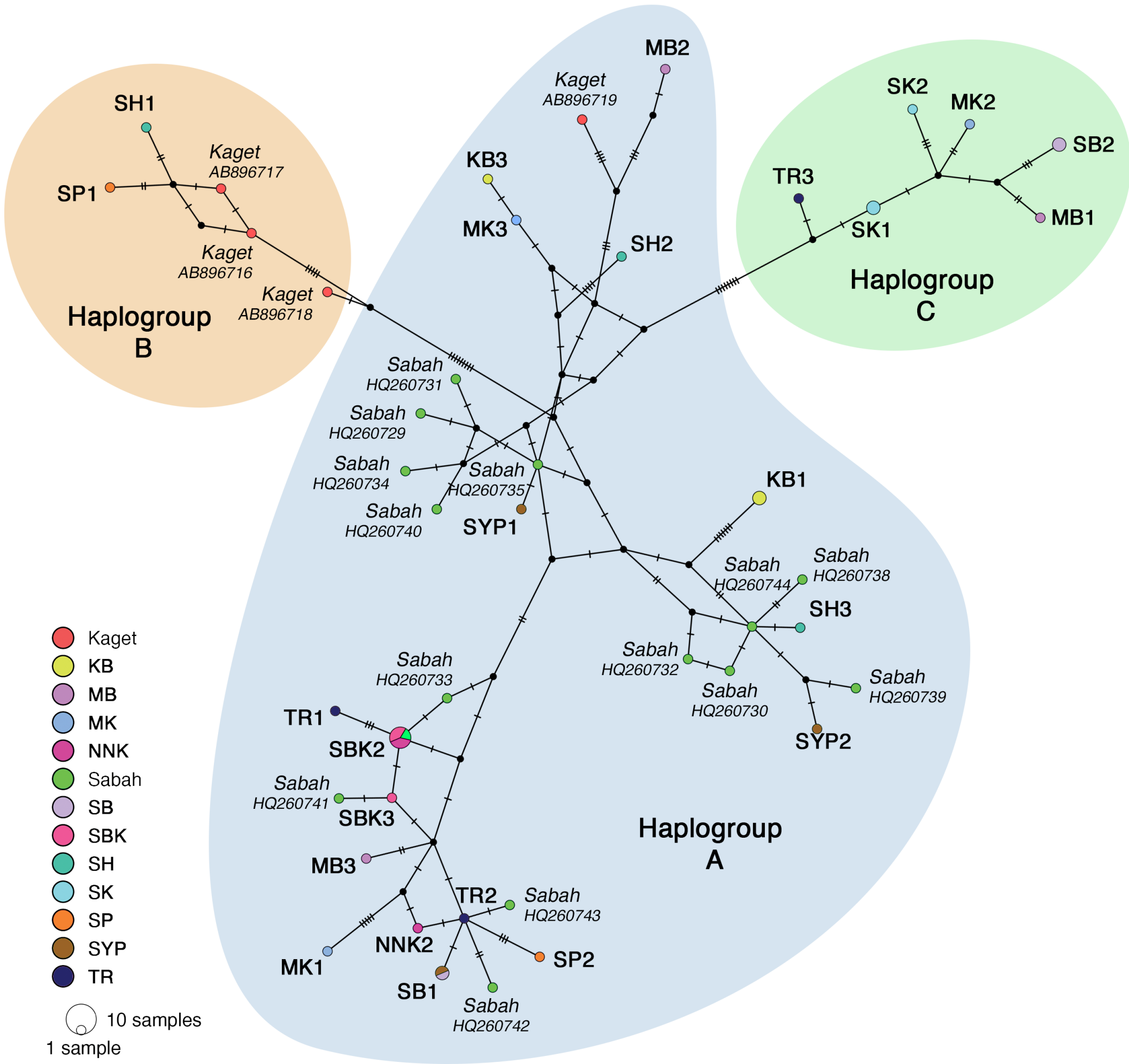

Fig. S3 Median-joining network of the mitochondrial control region for 51 *Nasalis larvatus* sequences of Dataset 2 (32 study samples, 4 from Pulau Kaget, and 15 published Sabah reference sequences), covering a 283-bp segment and resolving 43 haplotypes. Nodes are labeled with sample name or accession number and scaled by sequence count; hatch marks indicate mutational steps; small black nodes represent inferred median vectors. Background shading highlights the three haplogroups as shown in **Fig. 4**.

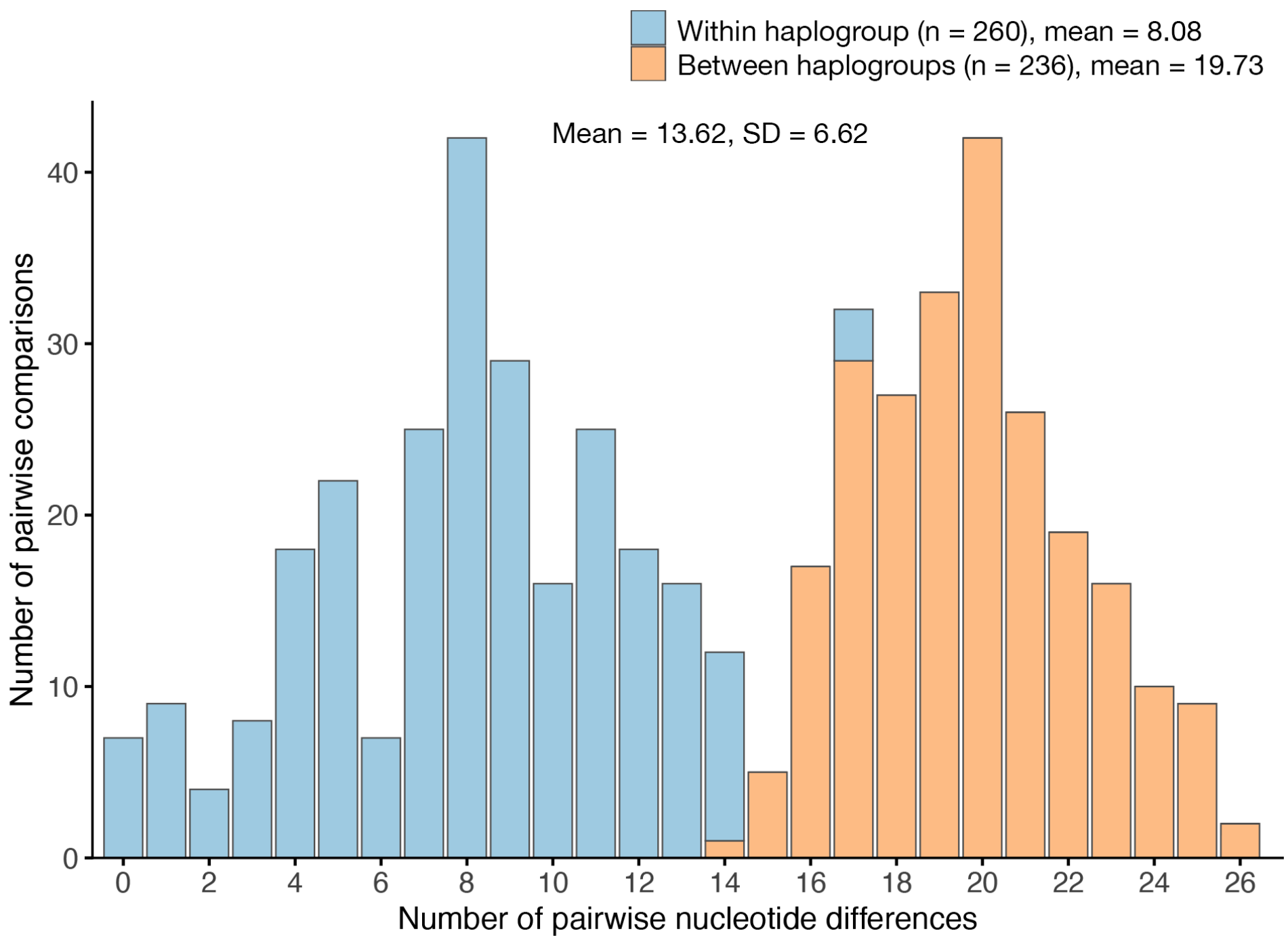

Fig. S4 The distribution of pairwise nucleotide differences among 32 *Nasalis larvatus* control-region sequences from eastern Borneo (496 comparisons) shows a bimodal pattern. Bars are shaded to indicate if the compared sequences belong to the same haplogroup (blue) or different haplogroups (orange). No demographic model has been fitted to these data.

Table S1. Distribution of mitochondrial control-region haplotypes among the 11 sampled sites of *Nasalis larvatus* in eastern Borneo.

Haplotype	NNK	SBK	SYP	TR	SB	SK	MK	MB	KB	SH	SP	Total
H1	0	0	0	0	0	0	0	0	0	1	0	1
H2	0	0	0	0	0	0	0	0	0	0	1	1
H3	0	0	0	0	2	0	0	0	0	0	0	2
H4	0	0	0	0	0	1	0	0	0	0	0	1
H5	0	0	0	0	0	0	0	1	0	0	0	1
H6	0	0	0	0	0	0	1	0	0	0	0	1
H7	0	0	0	1	0	0	0	0	0	0	0	1
H8	0	0	0	0	0	2	0	0	0	0	0	2
H9	0	0	1	0	0	0	0	0	0	0	0	1
H10	0	0	0	0	0	0	0	0	0	1	0	1
H11	0	0	0	0	0	0	1	0	0	0	0	1
H12	0	0	0	0	0	0	0	0	1	0	0	1
H13	0	0	1	0	0	0	0	0	0	0	0	1
H14	0	0	0	0	0	0	0	0	0	1	0	1
H15	0	0	0	0	0	0	0	1	0	0	0	1
H16	0	0	0	1	0	0	0	0	0	0	0	1
H17	0	1	0	0	0	0	0	0	0	0	0	1
H18	2	1	0	0	0	0	0	0	0	0	0	3
H19	0	1	0	0	0	0	0	0	0	0	0	1
H20	0	0	0	0	0	0	0	1	0	0	0	1
H21	1	0	0	0	0	0	0	0	0	0	0	1
H22	0	0	0	1	0	0	0	0	0	0	0	1
H23	0	0	1	0	1	0	0	0	0	0	0	2
H24	0	0	0	0	0	0	0	0	0	0	1	1
H25	0	0	0	0	0	0	1	0	0	0	0	1
H26	0	0	0	0	0	0	0	0	2	0	0	2
**Total**	**3**	**3**	**3**	**3**	**3**	**3**	**3**	**3**	**3**	**3**	**2**	**32**

Cell entries indicate the number of individuals at each locality carrying each haplotype. Sites are ordered from north to south along the eastern Borneo coast (NNK, Nunukan; SBK, Sembakung; SYP, Sesayap; TR, Tarakan; SB, Sungai Birang; SK, Sungai Kelindakan; MK, Muara Kaman; MB, Muara Badak; KB, Kutai Barat; SH, Sungai Hitam; SP, Sungai Parit). Haplotype numbering corresponds to the unique haplotype identifiers H1–H26 (GenBank accessions LC911855–LC911880).

Table S2. Fecal sample mass, DNA yield, and purity for the 32 *Nasalis larvatus* samples from eastern Borneo.

Sample	Locality	Fecal mass (g)	DNA concentration (ng/µl)	A260/A280
**NNK1**	Nunukan	0.58	78	1.75
**NNK2**	Nunukan	0.54	76	1.81
**NNK3**	Nunukan	0.50	31	1.86
**SBK1**	Sembakung	0.55	9.6	1.76
**SBK2**	Sembakung	0.52	22	1.74
**SBK3**	Sembakung	0.53	54.5	1.82
**SYP1**	Sesayap	0.56	40	1.70
**SYP2**	Sesayap	0.55	41	1.75
**SYP3**	Sesayap	0.54	36	1.85
**TR1**	Tarakan	0.57	200	1.77
**TR2**	Tarakan	0.55	87	1.85
**TR3**	Tarakan	0.56	211	1.80
**SB1**	Sungai Birang	0.56	63.5	1.81
**SB2**	Sungai Birang	0.51	23	1.28
**SB3**	Sungai Birang	0.53	54.5	1.70
**SK1**	Sungai Kelindakan	0.52	49	1.69
**SK2**	Sungai Kelindakan	0.59	21.5	1.75
**SK3**	Sungai Kelindakan	0.57	26	1.91
**MK1**	Muara Kaman	0.55	45.5	1.78
**MK2**	Muara Kaman	0.57	23	1.47
**MK3**	Muara Kaman	0.59	48.5	1.70
**MB1**	Muara Badak	0.44	43.5	1.81
**MB2**	Muara Badak	0.54	53	1.90
**MB3**	Muara Badak	0.52	33.5	1.76
**KB1**	Kutai Barat	0.53	25	1.79
**KB2**	Kutai Barat	0.53	41	1.86
**KB3**	Kutai Barat	0.53	86.5	1.84
**SH1**	Sungai Hitam	0.54	32	1.83
**SH2**	Sungai Hitam	0.55	18	1.83
**SH3**	Sungai Hitam	0.57	78	1.83
**SP1**	Sungai Parit	0.50	3	1.62
**SP2**	Sungai Parit	0.55	7.1	1.68
**Mean**		0.54	51.9	1.76
**Range**		0.44–0.59	3.0–211.0	1.28–1.91
